# Executive Functioning: Assessing the Role of Perceived Paranormal Ability

**DOI:** 10.3389/fpsyg.2021.798283

**Published:** 2021-12-23

**Authors:** Kenneth Graham Drinkwater, Neil Dagnall, Andrew Denovan, Andrew Parker, Álex Escolà-Gascón

**Affiliations:** ^1^Department of Psychology, Manchester Metropolitan University, Manchester, United Kingdom; ^2^Adelphi Values Ltd, Bollington, United Kingdom; ^3^Blanquerna Foundation, Barcelona, Spain

**Keywords:** paranormal ability, paranormal belief, executive functions, self-report measures, multivariate analysis of variance

## Abstract

This study examined whether scores on self-report measures of executive functions varied in accordance with level of self-professed paranormal ability. The investigators compared three groups varying in attribution of paranormal facilities: practitioners (Mediums, Psychics, Spiritualists and Fortune-Tellers), self-professed ability and no ability. Consistent with recent research on cognitive-perceptual factors allied to delusional formation and thinking style, the researchers anticipated that practitioners would score higher on paranormal belief and self-reported executive function disruption. Correspondingly, the investigators also hypothesised that the self-professed ability group would demonstrate greater belief in the paranormal and higher levels of executive function disruption than the no ability group. A sample of 499 (219 males, 279 females) respondents completed the measures online. Multivariate analysis of variance (MANOVA) found a large effect size, alongside significant differences on all variables apart from Cognitive Reappraisal. Pairwise comparisons indicated that Paranormal Belief increased as a function of level of ability; practitioners scored higher than self-professed, who in turn scored higher than the no ability group. For executive functioning, significant differences emerged only for the no ability vs. self-professed ability and no ability vs. practising groups. Collectively, outcomes indicated that perception of ability, regardless of intensity of paranormal conviction, influenced subjective appraisal of executive functions. Failure to find consistent differences between practitioner and self-professed ability groups suggested that discernment of ability was sufficient to heighten awareness of executive functioning disruptions.

## Introduction

Irwin and Watt (2007, p. 1) define paranormal experiences as ‘apparent anomalies of behaviour and experience that exist apart from currently known explanatory mechanisms that account for organism-environment and organism–organism information and influence flow’. The study of personal supernatural encounters is important because they represent a relatively common feature of existence (e.g., Tenhaeff, 1972; Schouten, 1994; Dagnall et al., 2016). Furthermore, they can have a profound impact on experiments (Schmied-Knittel and Schetsche, 2005; Laythe et al., 2021). However, despite their importance, paranormal experiences in comparison to beliefs remain relatively under researched. Moreover, investigators have historically focused on only narrow operationalisations, such as subjective paranormal experiences (SPEs), which denote an individual’s readiness to ascribe supernatural causation to an event or occurrence (Glicksohn, 1990).

Nonetheless, SPEs are a useful index because that they recognise the phenomenological significance of explicit individual encounters (Drinkwater et al., 2013) and attributional processes (Irwin et al., 2013; Lange et al., 2019; Laythe et al., 2021). However, SPEs are limited as they focus on singular incidents, whereas analysis of interviews indicates that many supernatural occurrences involve multiple, sustained events and are dispositional in nature. Furthermore, accounts are often vague and uncertain; being defined as strange and unusual and only possibly, paranormal in origin (Drinkwater et al., 2017). In this context, SPEs predominantly index spontaneous, important instances that are externally generated, beyond individual control (i.e., receptive rather than productive) and self-labelled. Thus, SPEs as a unit of measurement, often conflate person-centred (internal) and situational (external) factors. Consequently, they are largely insensitive to perceived possession of paranormal abilities, which belong to the percipient, manifest in a variety of ways and endure over time. Consistent with this delineation, ability-based accounts are often vague, ill-defined and draw on a range of unsubstantiated, anecdotal evidence. For these reasons, ability-based experiences differ qualitatively and quantitatively to regularly reported SPEs (i.e., ghosts/hauntings and extrasensory perception).

Noting these factors, Drinkwater et al. (2021a,b) used self-professed paranormal ability as an index of experience. Although researchers have under investigated the role of facility, measurement instruments have historically recognised the importance and independence of ability, belief and experience. Notable examples being the Anomalous Experiences Inventory (AEI; Gallagher et al., 1994), the Multivariable Multiaxial Suggestibility Inventory-2 (MMSI-2; Escolà-Gascón, 2020a; Escolà-Gascón et al., 2021) and the Australian Sheep Goat Scale (ASGS; Thalbourne and Delin, 1993). The AEI measures Anomalous/Paranormal Experiences alongside Beliefs, Fear and Ability. The MMSI-2 examines Anomalous Perceived Phenomena (APP) in conjunction with 12 cognitive and personality scales, which include belief in ‘psychic abilities’. The ASGS, which remains a widely used tool for assessing paranormal credence, includes items assessing ability (e.g., ‘I believe I have marked psychokinetic ability’), experience (e.g., ‘I believe I have had at least one experience of telepathy between myself and another person’) and belief (e.g., ‘I believe in the existence of psychokinesis’).

The inclusion of ability alongside experience and belief within the AEI and the ASGS reflects that facility is a core component of paranormal ideation that merits consideration. In support of this supposition, examination of the constructs reveals that while they overlap, they are discrete and discernible. Conceptually, ability denotes perceived possession and enactment of paranormal powers, experience refers to the ascription of paranormality to an event or occurrence, and belief is faith in the existence of supernatural forces and/or abilities. Thus, although individuals often predicate their alleged ability on possible experiences, ability and experience are different constructs. For instance, correlations between AEI subscales reveal only medium-large associations (Drinkwater et al., 2021a). Moreover, ability (vs. experience and belief) is differentially associated with other variables. For example, ability does not correlate with neuroticism and general sensation seeking, whereas experience and belief are positively related (Gallagher et al., 1994).

The advantage of an ability-based measure is that it recognises that some individuals believe they possess enduring supernatural faculties. Moreover, studies have also reported associations between self-professed paranormal powers and cognitive-perceptual characteristics. For instance, Parra and Carlos Argibay (2012) found that alleged psychics (vs. controls) scored higher on dissociation, absorption and fantasy proneness. Building on this work, Drinkwater et al. (2021a) investigated whether scores on cognitive-perceptual factors related to subclinical delusion formation and thinking style differed as a function of self-professed paranormal ability. They found that, compared with no and self-professed ability conditions, paranormal practitioners (i.e., Mediums, Psychics, Spiritualists and Fortune-Tellers) scored higher on proneness to reality testing deficits, emotion-based reasoning and paranormal belief. Analysis revealed similar differences between the self-professed and no ability conditions. These outcomes indicated that variations in self-perceived supernatural powers were associated with differences in cognitive-perceptual style.

This notion is commensurate with research that reports individuals scoring high on cognitive-perceptual factors allied to belief in the paranormal demonstrate subtle impairments in executive function, working memory and attention (Noguchi et al., 2008). Particularly, studies using non-clinical student populations, note inverse relationships between schizotypy and neurocognitive functioning (e.g., Jahshan and Sergi, 2007). Functions include executive functioning as assessed by the Wisconsin Card Sorting Test (i.e., abstract reasoning ability and capacity to shift cognitive strategies in response to changing external demands/rule changes; Daneluzzo et al., 1998), spatial working memory (Park and McTigue, 1997) and sustained attention (Gooding et al., 2006).

Advancing this work, the present paper examined relationships between self-professed paranormal ability and executive functions. Executive functions broadly denote cognitive processes that comprise top-down control (Burgess and Simons, 2005; Diamond, 2013). These include short-term storage and active manipulation of information within current attentional focus (working memory), selection of specific information amidst other data for subsequent processing (interference control), self-control and resistance to acting on impulse (inhibition) and processing that provides a basis for mentation and behaviour outside pre-established frameworks (Diamond, 2013).

Although previous work has explored paranormal belief and executive functions (e.g., Wain and Spinella, 2007), few studies have considered experience, especially self-professed ability. Delorme et al. (2013) conducted a relevant study with mediums. In one experiment, the investigators measured physiological responses during a psychic reading. Analyses revealed one medium displayed a decrease in prefrontal midline theta waves when making accurate responses (i.e., corresponding to the individual; Delorme et al., 2013). Functionally, this may have resulted from a decrease in executive or working memory processing, which was concomitant with the medium entering a ‘receptive mental state’, where the transfer of anomalous information could occur. This elucidation is speculative because it derived from one medium. An alternative explanation is that executive control processes facilitate the voluntary capacity to enter trance-like states that requires intentional action that manifests as prefrontal activations (e.g., Mainieri et al., 2017). In support of the supposition that activation of executive functions is associated with sense of control, Escolà-Gascón (2020b) conducted an experiment with purported mediums. In relation to success on experimental tests of anomalous information reception, the investigation reported a negative correlation between altered states of consciousness and suggestion.

Additionally, work by Delorme et al. (2018) tested mediums on a task requiring dead or alive decisions for unknown faces. Some mediums performed above chance, which was associated with right hemisphere parieto-occipital activations. However, this activity was more likely associated with attentional modulation than executive functioning. Other research using fMRI has discovered activations in sensory cortical regions during mediumistic trance-like states (Mainieri et al., 2017). These related to mediums reporting vivid visual and auditory spirits. However, the researchers also observed neural changes during imagined (non-mediumistic) trance states. Thus, alterations did not necessarily distinguish anomalous from non-anomalous states. Mainieri et al. (2017) also found frontal activations in mediumistic trance-like states. These were primarily in orbitofrontal regions rather than those more directly allied to executive functioning and likely reflected processes promoting sensory integration and evaluation.

An alternative approach to considering the role of executive functioning in anomalous states has used individuals who claim to have experienced spirit possession. Although the existence of different types of spirit possession (Bourguignon, 1976) complicates evaluation of cognitive-neural substrates, the distinction between intermittent and ‘transitory dissociative’ is useful in this context (Al-Adawi et al., 2001). The former denotes instances where a spirit has taken over an individual, whereas the latter refers to a brief state attendant with stressful events. Al-Adawi et al. (2001) found that the intermittent group (vs. transitory) scored lower on executive functioning (i.e., Wisconsin card sorting test, verbal fluency, trail making and the tower of London test). These finding contradict previously outlined work with mediums, which concluded that changes in neural activity signified increased executive control. This discrepancy may arise from the involuntary nature of spirit possession compared to the control mediums exercise over their trance-like states. Other work has shown that non-psychotic individuals, who claim anomalistic experiences, demonstrate lower performance on tests of executive function compared to controls (Powers et al., 2017).

### Present Study

This study extended preceding research by exploring whether scores on self-report measures of executive functioning varied as a function of self-professed paranormal ability. To allow comparisons with prior investigations on cognitive-perceptual factors, the present study used the same range of paranormal practitioners (i.e., Mediums, Psychics, Spiritualists and Fortune-Tellers) and categories (i.e., no ability vs. self-professed ability vs. practitioners) as Drinkwater et al. (2021a). Consistent with the outcomes of Drinkwater et al. (2021a), the researchers hypothesised that paranormal practitioners would score higher on paranormal belief and report greater levels of executive functioning disruption than the ability group, who correspondingly would score higher on measures than the no ability condition.

## Materials and Methods

### Respondents

Four hundred and ninety-nine respondents participated (Mean age, *M*) = 40.33 years, SD = 16.94, range 22–87. In terms of gender, 219 respondents were males (44%), *M* = 41.06 years, SD = 18.02, range 18–87; and 279 were females (56%), *M* = 39.83 years, SD = 16.05, range 18–75. To ensure there was a range of ability within sample data the investigators recruited the sample in two phases. The first targeted contacts acquired through the Paranormal Society and research projects. Since this convenience sample yielded only 152 respondents. The second used Bilendi, a social research company that provides participant panels for research and marketing. Bilendi samples have previously featured in published studies and reports (e.g., Lippke et al., 2021; van Schalkwyk et al., 2021). Accordingly, academics acknowledge the organisation as a source of quality samples. Moreover, panel data generally is comparable to that collected *via* traditional means in terms of reach and diversity (Kees et al., 2017). The researchers requested a Unite Kingdom-based representative sample comprising respondents aged 18 years and over.

### Measures

#### Self-Professed Paranormal Ability

Items assessed whether respondents believed that they possessed paranormal abilities (i.e., Spiritualist, Psychic, Medium and Fortune-Teller) and determined if they were practicing practitioners. The selected domains characterised primary, receptive components of paranormality (Dagnall et al., 2010c, 2011). To ensure that answers represented conceptual classifications, a clear explanation prefaced each ability (e.g., ‘Psychics perceive energy left behind from people who have died’). Respondents indicated the degree to which they believed they possessed facilities on a scale ranging 0 (no) to 100 (certain) and whether they were paranormal practitioners using a dichotomous ‘Yes/No’ option.

#### Executive Functions

Self-report instruments assessed a range of executive functions (i.e., general, memory and decision-making; see Drinkwater et al., Under Review).

#### Webexec

The Webexec is a 6-item instrument that measures general executive functioning problems. The scale was designed for Internet-mediated research and has featured in published work (e.g., Friedman-Krauss et al., 2014). Items index a range of executive functions including sustaining focus, concentration, multitasking, maintaining a train of thought, task completion and impulsivity. Within the Webexec, items appear as questions (‘Do you find yourself acting on “impulse?” ’). Respondents indicate answers on a 4-point scale ranging from 1 (no problems) to 4 (a great many problems). Summation of scores produces a total between 6 and 24; higher scores signify greater experience of executive functioning problems. The Webexec has established psychometric properties (i.e., satisfactory content validity and internal reliability; Buchanan et al., 2010).

#### Everyday Memory Questionnaire-Revised

The Everyday Memory Questionnaire-Revised (EMQ-R) consists of 13 items that index subjective memory failure in everyday life. Items present as behaviours (e.g., ‘Getting the details of what someone was told you mixed up and confused’) and respondents specify how frequently content has happened over the past month on a 5-point Likert scale. Scores range from 0 (once or less) to 4 (once or more in a day). The scale includes three subscales measuring Retrieval (memory failure), Attention Shifting (focus loss) and Factor 3 (visual reconstruction). The EMQ-R has good psychometric integrity (Royle and Lincoln, 2008).

#### The Working Memory Questionnaire

The Working Memory Questionnaire (WMQ) is a 30-item instrument that assesses working memory functioning. Scale items reference three facets: short-term storage (retention over a brief period), att43wention (mental slowness/fatigue, dual tasking and distractibility) and executive function (decision-making, planning and shifting). The WMQ presents items in the form of questions (e.g., ‘Do you feel that you tire quickly during the day?’) and respondents answer using a five-point Likert scale, which ranges from 0 (no problem at all) to 4 (very severe problem). Totalling of items produces a score between 0 and 120; higher scores reflect greater levels of working memory difficulties. The WMQ is an established, validated, robust psychometric instrument (Vallat-Azouvi et al., 2012).

#### Decision Making Questionnaire

The Control (e.g., ‘Do you enjoy making decisions?’) and Instinctiveness (e.g., ‘Do you rely on “gut feelings” when making decisions?’) subscales of the Decision Making Questionnaire (DMQ) evaluated decision-making efficacy (French et al., 1993). In combination, these dimensions assess impulsiveness (impetuosity), an important aspect of executive functioning. DMQ items appear as questions, and respondents answer *via* a six-point Likert scale ranging from 1 (Very infrequently or never) to 6 (Very frequently or always). French et al. (1993) validated the DMQ, and the instrument has featured in academic research (Douse and McManus, 1993; Kumar and Gupta, 2017).

### Other Measures

#### Emotion Regulation Questionnaire

The Emotion Regulation Questionnaire (ERQ; 10-items) assesses trait emotion regulation strategies. It comprises two subscales: cognitive reappraisal (e.g., ‘When I want to feel more positive emotion, I change the way I’m thinking about the situation’) and expressive suppression (e.g., ‘I keep my emotions to myself’). Respondents indicate the extent to which they agree with statements using a seven-point Likert scale 1 (strongly disagree) to 7 (strongly agree). The ERQ possesses robust psychometric properties (Gross and John, 2003).

### Belief in the Paranormal

#### Revised Paranormal Belief Scale

The Revised Paranormal Belief Scale (RPBS) is a 26-item instrument that assesses the extent to which individuals endorse the existence of paranormal phenomena (Tobacyk, 2004). Since its development, the scale has become the predominate measure of paranormal belief. Item content appears as statements. Participants respond *via* a seven-point Likert scale by selecting a choice ranging from 0 (strongly disagree) to 6 (strongly agree; Lange et al., 2000). Summation of items produces a score ranging from 0 to 156. Higher scores denote greater levels of paranormal belief. The RPBS possesses well-established measurement properties (i.e., validity and reliability; Drinkwater et al., 2017).

### Procedure

Respondents accessed the Participant Information Sheet (PIS) *via* a web link. The PIS outlined the study background then asked respondents to provide informed consent. Accepting participants then progressed to the instructions. These requested that respondents carefully read and attempt all items, advance through sections in their own time and answer openly and honestly. The study materials comprised sections on demographics (i.e., age and preferred gender), paranormal ability, executive functioning and belief in the paranormal. At the conclusion of materials, respondents were debriefed.

To address potential data contaminating factors the researchers employed procedural devices. Firstly, to prevent order effects, section and scale presentation rotated across participants. Secondly, to negate the influence of social desirability the instructions stated that there were no correct responses. Finally, to counter common method variance (CMV; Spector, 2019), scale instructions differentiated between constructs by emphasising scale uniqueness (Podsakoff et al., 2003). This was important as the study used a cross-sectional design. Data collected at one point in time is susceptible to CMV because measure proximity can inflate perceived relationships between constructs under observation.

### Ethics Statement

The Faculty of Health, Psychology and Social Care Ethics Committee at Manchester Metropolitan University (October 2018; Project ID, 954) provided approval for a programme of study investigating factors associated with self-professed psychic ability/mediumship.

## Results

### Preliminary Analyses

Respondents were categorised into three groups based on level of self-professed ability (no ability vs. ability vs. practising). Practitioner numbers were relatively similar across specialism (Mediumship, Psychic, Spiritualist and Fortune-Tellers; Table 1).

**Table 1:** Frequencies (and percentages in brackets) of professed ability and practitioner groups.

						Ability Ratings			
	Ability		Status			Practising		Non-practising	
Practitioner Group	Yes	No	Practising	Non-practising	Total	*M*	SD	*M*	SD
Mediumship	243 (49)	256 (51)	64 (26)	179 (74)	243	78.91	28.01	42.85	27.95
Psychic	255 (51)	244 (49)	59 (23)	196 (77)	255	77.63	30.08	43.32	29.23
Spiritualist	233 (47)	266 (53)	55 (24)	178 (76)	233	74.00	26.64	45.51	29.59
Fortune-Teller	218 (44)	281 (56)	26 (12)	192 (88)	218	67.31	25.70	39.06	26.75

Practitioners (vs. non-practising) expressed greater confidence about their self-professed paranormal abilities. However, since practitioners frequently reported multiple abilities/services it was not possible to compare differences. In terms of practitioner services, *n* = 34, 35% offered 1; *n* = 28, 29% offered 2; *n* = 22, 23% offered 3; and *n* = 12, 13% offered 4. Due to this overlap, practitioner services were combined to produce an overall group (*n* = 96, 19%). Analysis then explored difference between practitioners (*n* = 96, 19%), self-professed abilities (*n* = 197, 40%) and respondents declaring no abilities (*n* = 206, 41%). Descriptive information for paranormal belief and measures of executive functioning appears in Table 2.

**Table 2:** Reliability, means and standard deviations for paranormal belief and neuropsychological measures as a function of ability.

Outcome variable	Reliability (*α*)	Ability					
		No ability (*n* = 206)		Ability (*n* = 197)		Practising (*n* = 96)	
		*M*	SD	*M*	SD	*M*	SD
Paranormal belief	0.96	45.55	28.36	83.17	26.30	101.54	18.24
Executive function	0.89	10.82	3.65	12.88	4.54	13.31	4.36
Working memory	0.96	23.20	20.25	39.98	24.71	41.41	28.78
Decision-making	0.72	26.56	5.04	25.76	5.57	28.98	6.09
Retrieval	0.91	6.56	5.78	10.17	7.31	8.58	7.76
Attention tracking	0.85	2.36	2.95	4.71	4.11	4.70	4.52
Factor 3	0.69	0.80	1.37	1.86	2.05	1.76	2.21
Cognitive reappraisal	0.63	27.20	5.28	26.53	5.83	27.45	5.79
Expressive suppression	0.56	18.15	3.59	18.34	4.15	19.88	4.29

### Ability Group Comparisons

Multivariate analysis of variance (MANOVA) assessed whether ability scores (no ability, self-professed ability and practitioners) differed significantly on study measures (i.e., paranormal belief, general executive function, working and everyday memory, decision-making and emotion regulation). Since Drinkwater et al. (Under review) observed experience-based differences on EMQ-R (Retrieval, Attention Tracking and Factor 3) and ERQ (Expressive Suppression and Cognitive Reappraisal) subscales were included within the analysis.

Prior to MANOVA, data screening occurred. This indicated that all values were acceptable; fell within the range of −2 to +2 (Byrne, 2010). Data points represent outliers if they possess a studentised residual >4 and a Cook’s distance >4/(*n* − *k* − 1), where *n* represents the sample size and *k* denotes the number of independent variables. Using these criteria, no outliers arose. However, Box’s test (i.e., assessment of homogeneity of variance-covariance matrices) was significant (244.54, *p* < 0.001). Accordingly, interpretation used Pillai’s criterion instead of Wilk’s lambda because it is a more robust index.

MANOVA produced a significant overall effect, Pillai’s criterion = 0.54, *F* (18, 978) = 20.13, *p* < 0.001. A large effect size occurred (η^2^ = 0.27) indicating differences in measures across ability groups. Univariate analyses indicated significant differences on all variables apart from Cognitive Reappraisal (Table 3). Small to medium effect sizes existed for all outcomes except paranormal belief (large effect).

**Table 3:** Analysis of variance (ANOVA) and pairwise comparison summary for outcome variables.

Variable	ANOVA		Pairwise comparison		
	Ability overall		No ability vs. Ability	No ability vs. Practising	Ability vs. Practising
	*F^df^* (Sig.)	*η* ^2^	Mean diff. (Sig.)	Mean diff. (Sig.)	Mean diff. (Sig.)
Paranormal Belief	188.20^2, 496^ (<0.001)	0.43	−37.62 (<0.001)	−55.98 (<0.001)	−18.36 (<0.001)
Executive Function	17.26^2, 496^ (<0.001)	0.07	−2.06 (<0.001)	−2.48 (<0.001)	−0.42 (1.00)
Working Memory	31.71^2, 496^ (<0.001)	0.11	−16.78 (<0.001)	−18. 21 (<0.001)	−1.43 (1.00)
Decision-Making	11.38^2, 496^ (<0.001)	0.04	0.80 (0.425)	−2.42 (0.001)	−3.22 (<0.001)
Retrieval	14.14^2, 496^ (<0.001)	0.05	−3.60 (<0.001)	−2.02 (0.050)	1.58 (0.186)
Attention Tracking	23.39^2, 496^ (<0.001)	0.09	−2.34 (<0.001)	−2.34 (<0.001)	0.01 (1.00)
Factor 3	18.89^2, 496^ (<0.001)	0.07	−1.06 (<0.001)	−0.95 (<0.001)	0.10 (1.00)
Cognitive Reappraisal	1.12^2, 496^ (<0.001)	0.01	0.66 (0.702)	−0.25 (1.00)	−0.92 (0.563)
Expressive Suppression	6.77^2, 496^ (<0.001)	0.03	−0.19 (1.00)	−1.73 (0.001)	−1.54 (0.006)

*η*^2^, partial eta-squared.

Pairwise comparisons (Bonferroni) revealed that Paranormal Belief increased as a function of level of ability; practitioners scored higher than self-professed, who scored higher than no ability (Table 3). For General Executive Functioning, Working Memory and Everyday Memory significant differences emerged for the no ability vs. ability and the no ability vs. practising comparisons. There were no differences between the ability conditions (ability vs. practising). For Decision Making and Expressive Suppression, analysis revealed significant differences between no ability vs. practising and ability vs. practising groups. Further contrast analysis (Table 4) indicated that for all measures, except for Decision Making and Cognitive Reappraisal, ability groups (self-professed ability and practitioners) reported greater executive functioning problems.

**Table 4:** Contrasts between no ability, ability and practising groups in relation to executive functioning.

Variable	Contrast (no ability vs. ability and practising)		
	*df*	*t* (Sig.)	Cohen’s *d*
Executive function	496	6.06 (*p* < 0.001)	0.55
Working memory	496	7.88 (*p* < 0.001)	0.72
Decision-making	496	1.59 (0.112)	0.15
Retrieval	496	4.52 (*p* < 0.001)	0.41
Attention tracking	496	6.85 (*p* < 0.001)	0.62
Factor 3	496	6.09 (*p* < 0.001)	0.56
Cognitive reappraisal	496	−0.39 (0.695)	0.04
Expressive suppression	496	2.60 (0.009)	0.24

## Discussion

Analysis revealed ability-related differences on several of the measures used in this study. Generally, perceived ability was associated with greater reported levels of Paranormal Belief and executive function disruption. However, practitioners (vs. self-professed) only scored higher on Paranormal Belief, Decision Making and Expressive Suppression. Collectively, these outcomes indicated that perception of ability, regardless of intensity of paranormal conviction, influenced subjective appraisal of executive functions. The failure to find consistent differences between the declared ability groups on executive function contrasted with Drinkwater et al. (2021a), who observed ability-related variations on cognitive-perceptual measures (proneness to reality testing deficits and emotion-based reasoning).

In the present study, the failure to find consistent differences between practitioners and those with self-professed ability is difficult to explain. The practitioners scored higher on Paranormal Belief and reported greater confidence in their alleged abilities. Commensurate with Drinkwater et al. (2021a), this suggests that the two groups differ in their perceptions of the paranormal. One possibility, based on the observed outcomes, is that discernment of ability was sufficient to heighten awareness of executive functioning disruptions. Tentatively, this could be an artefact of the scales used, which focus on degree of perceived disruption. In this context, the important distinction is between presence/absence of disruption rather than extent. The latter classification is likely lacking in discriminatory power because it is sensitive to subjective variations in ratings. Additionally, although no significant difference in executive functioning was found between the practitioners and self-professed group, the numerical difference was in the expected direction. Specifically, it was higher for the practitioner group. Given that the magnitude of the disparity in Paranormal Belief scores was smaller between these groups and the corresponding no ability group, then it is possible that the variation in Paranormal Belief was insufficient to detect any real underlying differences.

While the current paper was exploratory in nature, it is important to recognise limitations. One issue was the use of self-report measures. These rely upon the introspection of executive functioning and accordingly are susceptible to interpretative bias. A related concern with metacognitive assessments of processes is that higher-order functions may not be fully accessible to consciousness (Dagnall et al., 2008; Chan et al., 2015).

Concomitantly, heightened awareness of executive function disturbance may reflect disparities in cognitive focus rather than actual impairment. There is substantial evidence to support this notion. Specifically, both experience and belief in the paranormal correlate positively with variables associated with unusual perceptions and ideations (i.e., proneness to reality testing deficits, Dagnall et al., 2018; and schizotypy, Dagnall et al., 2010b). This coincides with a preference for intuitive, intra-psychic data (Dagnall et al., 2010a; Drinkwater et al., 2012). Hence, perceived executive function disruption in respondents professing paranormal abilities could merely reflect an internalised focus.

Additionally, collecting data at one point in time is potentially problematic because evaluations are recall based and susceptible to deviation as a function of accessibility, availability and context (e.g., Schwarz, 2007). Hence, subsequent studies should establish the veracity of intra-respondent judgments by taking multiple assessments at different time points. Although these may still not accord with performance on objective measures, compound ratings will provide a more robust index of individual subjective ratings. Moreover, researchers could compare these with proxy ratings. In the related area of prospective memory, proxy (vs. self-ratings) have proved invaluable since they often correlate more strongly with objective assessments (Arnold and Bayen, 2019). Nonetheless, to ensure that present outcomes accord with actual performance it is essential that subsequent work cross-tabulates rating-based work with objective measures (Buchanan et al., 2010). This is essential to collaborate the tentative findings of this study. Moreover, testing should also include a broader range of executive functions. In addition, although this study included explicit efforts to encourage response accuracy among the sample, the implementation of attention check items may have limited potential response error to a greater degree.

Finally, the present study used a narrow range of abilities and practitioners (Drinkwater et al., 2021a for a detailed discussion). While professions were commensurate with core paranormal domains, psychic occurrences and communication with the dead, these represented only a subsample of practitioners in areas allied to scientifically unsubstantiated beliefs. Additionally, asking practitioners to label their abilities is problematic because individuals often provide a range of overlapping but distinct services. In this context, future studies could use wider inclusive categories, based on alleged ability rather than service provided (i.e., productive vs. receptive), and assess a broader range of characteristics. The present study used confidence, which represents only one element that contributes to self-perception of capacity. Consideration of the general literature on perceived ability identifies other important factors, such as control, efficacy, motivation, success and commitment, that merit consideration. This is necessary since developing a fuller understanding of the psychological factors associated with self-perceived ability will inform subsequent studies and help to identify potential variables that influence high-order cognitive processes.

## Data Availability Statement

The raw data supporting the conclusions of this article will be made available by the authors, without undue reservation.

## Ethics Statement

The studies involving human participants were reviewed and approved by Manchester Metropolitan University Faculty of Health, Psychology and Social Care Ethics Committee. The patients/participants provided their written informed consent to participate in this study.

## Author Contributions

KD and ND provided the theoretical focus, developed content, and produced the initial article. KD was responsible for data collection and measurement selection. AD performed data analysis and wrote up the results. ÁE-G contributed further analytical advice and support. AP offered additional input on executive functions. All authors contributed to the article and approved the submitted version.

## Funding

This research was supported by the BIAL Foundation (Project Number 082/2018/G-07745). The funder was not involved in the study design, collection, analysis, interpretation of data, the writing of this article or the decision to submit it for publication.

## Conflict of Interest

AD was employed by Adelphi Values Ltd.

The remaining authors declare that the research was conducted in the absence of any commercial or financial relationships that could be construed as a potential conflict of interest.

## Publisher’s Note

All claims expressed in this article are solely those of the authors and do not necessarily represent those of their affiliated organizations, or those of the publisher, the editors and the reviewers. Any product that may be evaluated in this article, or claim that may be made by its manufacturer, is not guaranteed or endorsed by the publisher.
